# Acute Pulmonary Embolism Treated by Surgery and Complicated With a Pulmonary Artery Pseudoaneurysm: A Case Report

**DOI:** 10.7759/cureus.95642

**Published:** 2025-10-29

**Authors:** Arthur Dumont, Gaby Aphram, Francesca Chiara Della Casa, Christophe Beauloye, Diego Orbegozo

**Affiliations:** 1 Department of Cardiology, Université Catholique de Louvain, Brussels, BEL; 2 Department of Cardiothoracic Surgery, Cliniques Universitaires Saint-Luc, Brussels, BEL; 3 Department of Cardiovascular Intensive Care, Cliniques Universitaires Saint-Luc, Brussels, BEL

**Keywords:** clot in transit, endovascular embolization, patent foramen ovale, pulmonary artery embolization, pulmonary embolism, surgical embolectomy

## Abstract

Acute pulmonary embolism (PE) remains a relatively common pathology, with a high morbidity and mortality. Recommended treatment is based on anticoagulation, and thrombolysis is reserved for patients with hemodynamic instability. However, in some cases, these treatments are contraindicated or ineffective, and then other strategies are needed. Here, we present the case of a 48-year-old female patient presenting with a submassive PE showing a highly mobile intracardiac thrombus straddling through a patent foramen ovale. Considering the high risk of systemic and cerebral embolization with pharmacologic thrombolysis or with endovascular thrombectomy, we opted for a surgical pulmonary thrombectomy (SPT). This procedure was performed successfully but complicated by a left lower pulmonary artery pseudoaneurysm, probably related to the use of a Fogarty catheter for distal embolectomy. This complication was treated by interventional radiology with an endovascular embolization. Subsequent evolution was favorable under systemic anticoagulation, and the patient was discharged home on day 19. The ambulatory follow-up showed a complete recovery after three months. Nowadays, observed postoperative mortality and complication rates with SPT in the context of acute PE are acceptable and somewhat lower than predicted ones. Considering that SPT remains rarely used nowadays, but that good outcomes are frequently reported in recent literature, we decided to perform a brief literature review to highlight recent advances in this field.

## Introduction

Acute pulmonary embolism (PE) is the third leading cause of cardiovascular mortality, affecting all population groups, with high societal costs, morbidity, and mortality [1]. The annual incidence rates for PE are between 39 and 115 cases per 100,000 population [2]. We must highlight that around a third of patients die suddenly or within a few hours of the acute event, even before any therapy can be initiated. For those surviving this initial period, it is of capital importance to evaluate the exact impact of the acute PE on the cardiovascular system, as hemodynamic instability is associated with worse outcomes, including a higher risk of mortality [1,2]. Contemporaneous risk stratification uses hemodynamics, imaging, and laboratory parameters to classify patients into massive, submassive, or nonmassive PE, as proposed by the American Heart Association (AHA). This classification overlaps with the proposed one by the European Society of Cardiology (ESC) that classifies patients into high, intermediate, or low mortality risk PE, respectively [1]. 

The key treatment for most cases is based on therapeutic anticoagulation, which is effective and generally sufficient in most cases [1,2]. Major guidelines agree that in the presence of massive or high-risk PE, intravenous thrombolysis has demonstrated its efficacy, and then, it must be the first-line therapy of choice [1,2]. However, considering that this strategy is accompanied by a higher risk of bleeding and that it is sometimes contraindicated, other treatments are needed. During the last decades, we have seen an important evolution of other curative treatments such as the percutaneous and the surgical pulmonary thrombectomy (SPT) and supportive therapies, including different types of transient mechanical circulatory support devices [2,3].

Despite these advances, there is little data on the management of PE complicated by intracardiac thrombi, especially on the left side [4]. In our case, we present the management of a submassive PE complicated by an extensive and extremely mobile intracardiac thrombus, from the right to the left cavities, via a patent foramen ovale.

## Case presentation

A 48-year-old female patient was referred to the emergency department by her attending physician for dyspnea evolving over two weeks, associated with right leg painful edema. On the medical history, atypical, nonpleuritic chest pain and a dry cough were noted.

The patient was known for fibromyalgia, esophagitis, rheumatoid arthritis, hypothyroidism, endometriosis, and prediabetes. She had no history of smoking. Chronic medication included L-thyroxine, weekly tocilizumab, contraceptive therapy with nomegestrol, metformin, simvastatin, and esomeprazole.

Admission parameters showed a heart rate of 144 bpm, a respiratory rate of 22 bpm, a pulse oxygen saturation of 98%, a supine blood pressure of 111/82 mmHg, and a temperature of 37.7°C. Physical examination was unremarkable except for tachycardia and edema of the right lower limb with a positive Homans' sign. The ECG showed a sinus tachycardia with narrow QRS complexes, but with a right axial deviation and an S1T3 pattern.

Admission laboratory values are presented in Table 1, with high D-dimers, troponin I, and N-terminal pro-B-type natriuretic peptide (NT pro-BNP) values. A lower limb Doppler confirmed a deep vein thrombosis at the ostium of the right superficial femoral vein, and a CT pulmonary angiogram showed a large, proximal, and bilateral PE. Transthoracic echocardiography showed dilatation of the right cavities with severe impairment of right ventricular function, and a large thrombus in the right atrium impeding tricuspid valve dynamics. 

**Table 1:** Laboratory test results at admission AST: aspartate aminotransferase; ALT: alanine aminotransferase; aPTT: activated partial thromboplastin time; INR: international normalized ratio; PCO2: carbon dioxide pressure; PaO2: arterial oxygen pressure; FiO2: inspired oxygen fraction; NT-pro-BNP: N-terminal pro-B-type natriuretic peptide

Variable	Admission value	Reference values
Hemoglobin (g/dL)	12.8	12.2-15.0
Leukocytes (x10^3^/µL)	14.6	4.0-10.0
Neutrophils (%)	85	40-70
Platelets (x10^3^/µL)	163	150-450
C-reactive protein (mg/L)	48	<5
Creatinine (mg/dL)	1.06	0.6-1.2
Total bilirubin (mg/dL)	0.6	<1.2
AST (U/L)	30	13-35
ALT (U/L)	25	7-35
aPTT (sec)	26	24-36
INR	1.3	0.8-1.2
pH	7.46	7.35-7.45
PCO2 (mmHg)	24	35-45
PaO2 (mmHg)	67	74-108
PaO2/FiO2 ratio (mmHg/%)	340	>300
Lactate (mmol/L)	3.9	0.5-2.0
Troponin I (ng/L)	97.1	<45.1
NT-pro-BNP (pg/mL)	19975	<125
D dimer (µg/mL)	>4.0	<0.5
Fibrinogen (mg/dL)	333	200-400

**Figure 1:**
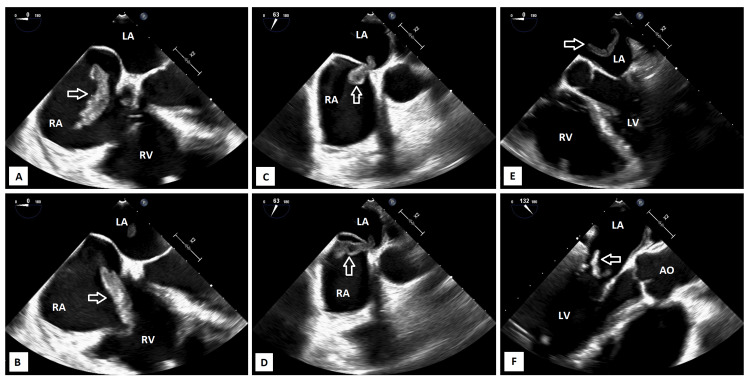
Large mobile embolus (white arrow) in the right atrium (A), moving through the tricuspid valve (B), straddling the foramen ovale (C and D), reaching the left atrium (E), and moving through the mitral valve (F) RA: right atrium; RV: right ventricle; LA: left atrium; LV: left ventricle; AO: aorta

At this point, the diagnosis of a submassive acute PE was retained, and the patient was admitted to the intensive care unit for close monitoring. Continuous intravenous unfractionated heparin was started as the initial treatment, considering its short half-life and the risk of patient deterioration. On day two, hemodynamics remained uncompromised, but an echocardiographic deterioration was noted, with increasing pulmonary pressures (>60 mmHg), a floating serpiginous clot of 8 x 49 mm in the right atrium, and suspicion of migration into the left cavities. A transesophageal echocardiography was performed, which confirmed a large bi-atrial mobile thrombus straddling the patent foramen ovale and moving through the tricuspid and mitral valve planes (Figure 1).

Considering the high risk of systemic and cerebral embolization with pharmacologic thrombolysis or with an endovascular thrombectomy, it was decided by our multidisciplinary acute heart team (composed of a senior cardiologist, an interventional cardiologist, an intensive care specialist, and a cardiac surgeon) to proceed with a surgical treatment. It was critically important that all data (medical history, hemodynamics, and imaging studies) pointed to an acute PE, without any sign suggesting a chronic thromboembolic pulmonary disease. 

**Figure 2:**
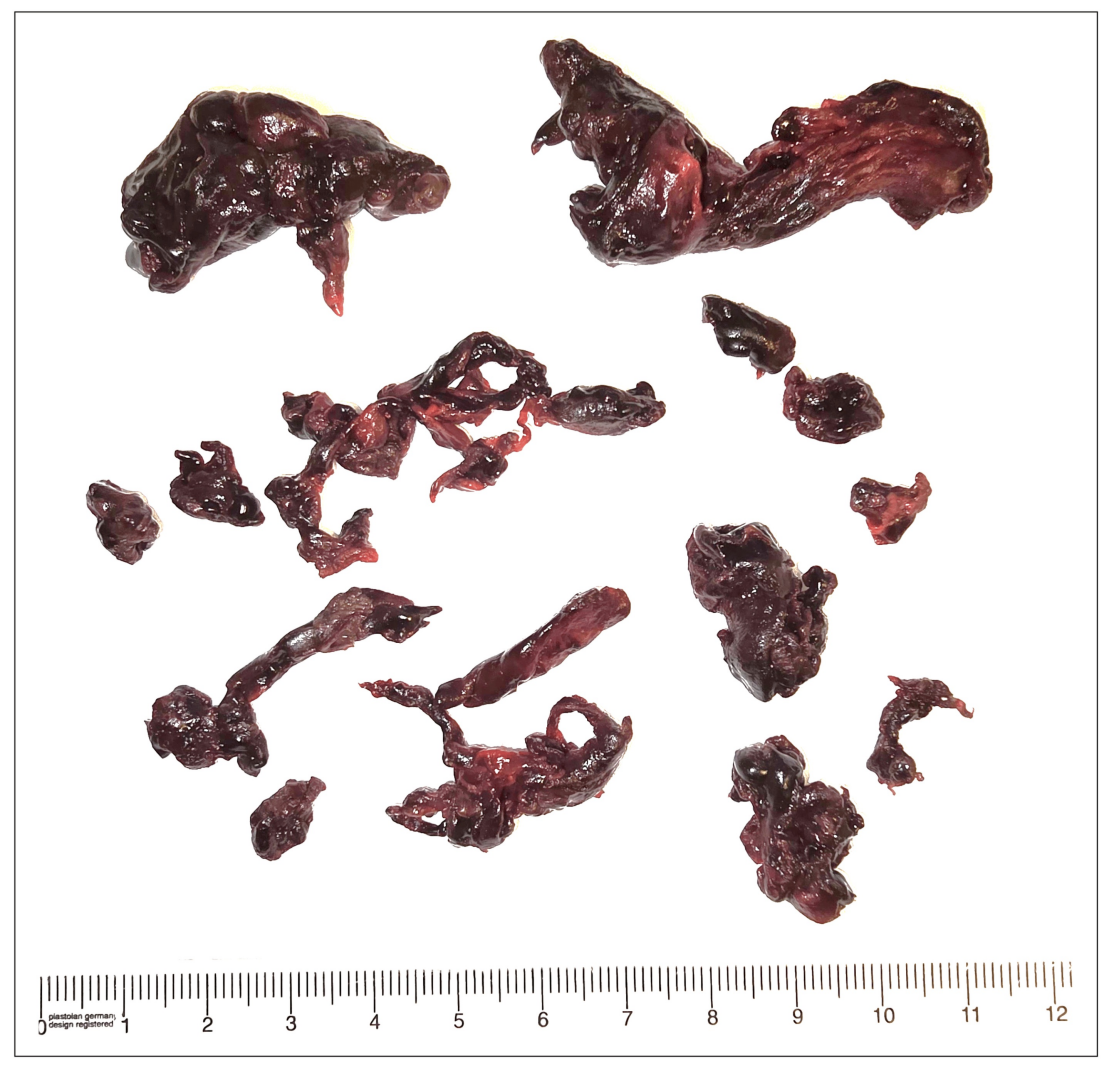
Surgically extracted cardiac and pulmonary thrombi

On day three, a bi-atrial SPT with an extensive bilateral pulmonary and intracardiac embolectomy was performed (Figure 2). This procedure was performed under cardiopulmonary bypass (CPB) with a total CPB time of 112 minutes and an aortic cross-clamping time of 80 minutes. Intracardiac thrombi were removed through the right atrium with a trans-septal approach for the left atrium. Thereafter, the interatrial defect was repaired. Then, the pulmonary trunk and both pulmonary arteries were explored to remove all the clot burden. A Fogarty catheter was used for distal embolectomy. The SPT was completed without any intraoperative complications. 

**Figure 3:**
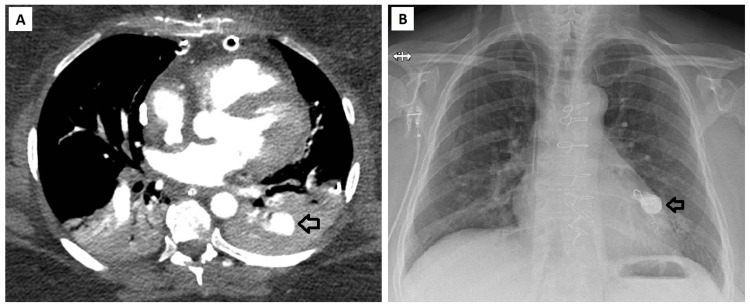
Pseudoaneurysm in the left lower pulmonary artery (black arrow) before (A) and 12 days after endovascular embolization (B)

On day four, hemodynamics remained stable but hypoxemia persisted, and a CT pulmonary angiogram showed a 2 cm pseudoaneurysm in the left lower lobe, very likely secondary to the use of the Fogarty catheter. Considering the high risk of spontaneous rupture, massive bleeding, and death if left untreated, it was decided to treat it immediately with an endovascular embolization. Interventional radiology securized the pseudoaneurysm by injecting coils and biological glue, and the procedure was completed without complications (Figure 3).

The subsequent evolution was favorable and uncomplicated, and the patient was able to return home on day 19. The ambulatory follow-up showed a complete echocardiographic recovery after one month and a normal lung V/Q scan after three months. Her clinical status remained normal nine months later, under therapeutic anticoagulation with apixaban. 

## Discussion

Contemporaneous treatment for acute PE varies between anticoagulation, thrombolysis, and hemodynamic support based on the degree of cardiovascular instability, but other strategies are needed when these therapies are contraindicated or insufficient [1,2]. Frequently, it is the risk of excessive bleeding that obligates an alternative treatment, but in our case, we were mainly concerned about the risk of systemic embolization of the clot in transit through the foramen ovale.

The proposed alternatives by the ESC include percutaneous and surgical thrombectomies in high-risk PE when thrombolysis has failed or is contraindicated [3], but those are rarely performed. In a large database in the UK, an SPT was used only in 256 patients during a period of 22 years in 33 hospitals, which represents around one patient every three years in each center [5]. In our institution, it is also an unusual procedure, but in our case, we opted for the surgical approach, considering that alternative options had a high risk of systemic clot embolization. This complex decision was taken by our acute heart team, a multidisciplinary group of specialists equivalent to the PE response team (PERT) proposed by current guidelines [1,2]. It has been repeatedly shown that mortality rates can be lowered (sometimes by a half) when PERTs are created in tertiary centers [1]. 

Surgical management was first described in 1908 by Trendelenburg, yet it was not until 1924 that his pupil Kirschner performed a successful procedure [6]. However, the modern surgical technique was improved with the introduction of CPB, and in 1961, John Gibbon started a new era with a significant reduction in the intraoperative mortality [3]. Today, the procedure is performed via median sternotomy, with bypass surgery and cardioplegia.

Moreover, there were considerable advances in CPB technology, with more biocompatible materials, but also including the possibility of parallel right-sided mechanical assistance and venoarterial extracorporeal membrane oxygenation (ECMO), leading to better survival rates after PE [1,3,7]. This scenario has renewed interest in SPT for massive and submassive PE.

Mortality has clearly decreased over the last decades in patients following surgical pulmonary thrombectomies, as shown by recently published literature reviews [3,7,8]. First, intraoperative mortality is between 2.7% and 4.0%, at some point comparable to urgent coronary artery bypass graft surgery [3,8]. Second, reported overall in-hospital mortality in different literature reviews is between 16% and 22%, but with a consistent trend toward lower values during the last decades [7,8].

A recent report of the literature evaluating a very large database in the USA of more than 1,000 hospitals has shown that between 2003 and 2014, there were 1,916,793 diagnoses of PE. Among them, only 3,486 patients underwent an SPT (thus, only 0.2% of the population). More interestingly, the observed in-hospital mortality was 14% in the more recent period between 2009 and 2014 [9].

Another recent observational study in the USA has reported an in-hospital mortality of 11.7% after SPT for acute PE, with 23.7% and 9.1% for massive and submassive PE, respectively [6]. This observation is remarkable considering recent observational data, including all types of patients with PE: the observed in-hospital mortality is around 28.3% with massive PE [10] and between 4.0% [11] and 6.7% [12] in submassive PE (whereas the predicted mortality rate was between 3% and 15%).

SPT is usually considered as a rescue strategy for PE, but it is better to say that lower mortalities have been described in specialized centers that consider it as a regular strategy in patients with intermediate or high risk of mortality. A recent Chinese cohort of 41 patients, where only 15 had contraindications for thrombolysis, reported a 7.3% in-hospital mortality [13]. A recent American cohort of 55 patients reported a 7% in-hospital mortality (even if predicted mortalities were higher) with a 100% survival in patients with sub-massive PE [14]. The best reported outcomes come from a cohort of 44 patients in the USA, where 79.5% had submassive PE, and reported a 2.3% in-hospital mortality [15]. Our center does not usually consider an SPT as the first-line treatment for PE, but this data suggests that we would consider it earlier in some specific cases. 

Early mortality in PE is mainly explained by acute right ventricular failure, and SPT can achieve complete or very important removal of the clot burden [3]. Different studies have shown that after surgery, there is a rapid and persistent improvement in the right ventricular function [13,14]. Our patient presented a severe impairment of right ventricular function with an acute pulmonary hypertension that resolved rapidly and persistently after the SPT.

Certainly, an optimal selection of patients is crucial before proceeding with an SPT. Usually, this strategy is chosen either when there are contraindications or treatment failures with other therapies (thrombolysis or catheter-guided thrombectomy), or when a cardiac surgery is needed for another reason (intracardiac thrombi, etc.) [7,16]. Worst outcomes have been observed in patients who needed cardiopulmonary resuscitation (CPR) before the surgery, in patients with a failed thrombolysis, in those with chronic pulmonary thromboembolic disease, or in cases of poor expected neurological outcome [1,3,6,14]. All these elements must be taken into consideration when determining which patients are suitable candidates for an SPT. Our patient did not present any of them.

Different complications have been described after an SPT. Pulmonary bleeding has been reported in around 10%, wound complications in around 7%, and surgical site bleeding in around 5% of cases [7,8,13]. Indeed, we observed some bloody endotracheal tube secretions during the first days, but in small quantities, and they resolved spontaneously without any specific treatment. However, our patient developed a pseudoaneurysm of the left pulmonary artery that was detected by a CT pulmonary angiogram performed one day after the surgery. Probably, the decision to use a Fogarty catheter to extract thrombi from distal pulmonary arteries was the cause of this complication. This complication has already been described, and the technical aspect of thrombotic extractions at this level is still controversial in the current literature [1]. Fortunately, it was successfully treated by percutaneous endovascular embolization, with no further repercussions.

Of course, an SPT (even with the complete extraction of the clot burden) does not obviate the need for further therapeutic anticoagulation, in accordance with current guidelines on PE [2]. Our decision was to start apixaban before hospital discharge, with a highly reassuring evolution thereafter and a complete normalization of the right ventricular function at one month. While this is a single-institution case report with nongeneralizable findings, our review may help PERTs better identify patients suitable for forgoing an SPT in the context of acute PE. 

## Conclusions

The efficacy and safety of SPT in the setting of acute PE have evolved favorably over the years, and it is nowadays an important option in selected cases. Acute PEs with coexisting intracardiac thrombi, especially on the left side, are rare and pose complex management challenges. This case demonstrates that a multidisciplinary team is crucial for achieving positive outcomes. Our report shows that early surgical management can be safe and effective, although it is currently reserved as a last-line treatment and primarily considered a rescue therapy. Our case also suggests that the use of Fogarty catheters for distal embolectomy during an SPT could lead to pulmonary artery pseudoaneurysms. Therefore, they should be avoided when possible. If this rare complication occurs, endovascular embolization remains the preferred treatment. 
